# Endovascular recanalization for acute posterior cerebral artery occlusion: a pre-specified secondary analysis of the ATTENTION, BAOCHE, and PLATO studies

**DOI:** 10.3389/fneur.2026.1820036

**Published:** 2026-07-03

**Authors:** Ziqiang Yu, Xiaomin Xue, Baichen Wang

**Affiliations:** 1 Department of Neurology, Tongde Hospital of Zhejiang Province, Hangzhou, China; 2 Institute of Basic Experiments, Zhejiang Academy of Traditional Chinese Medicine, Hangzhou, China

**Keywords:** endovascular thrombectomy, evidence gap, evidence synthesis, ischemic stroke, posterior cerebral artery

## Abstract

**Background:**

Acute posterior cerebral artery (PCA) occlusion causes disabling deficits, but evidence for endovascular therapy (EVT) is lacking. This gap exists between established efficacy of thrombectomy in anterior and posterior circulations.

**Objective:**

To evaluate evidence for PCA thrombectomy via a pre-specified synthesis of the ATTENTION, BAOCHE, and PLATO studies.

**Methods:**

Three evidence layers were integrated: (1) indirect supportive evidence from two RCTs on basilar artery occlusion (ATTENTION/BAOCHE) for posterior circulation EVT; (2) the targeted observational PLATO study for isolated PCA occlusion; and (3) connecting subgroup data. Efficacy and safety estimates (adjusted Risk Ratio [aRR] /adjusted Odds Ratio [aOR], 95% CI) were compared across studies.

**Results:**

ATTENTION (aRR 2.06, 1.46–2.91) and BAOCHE (aRR 1.81, 1.26–2.60) indirectly support the feasibility of the principle, enrolling relevant basilar tip occlusions (33% in ATTENTION, 12% in BAOCHE). PLATO provides direct target data, suggesting potential benefit for PCA occlusion (functional recovery aOR 1.13, 0.85–1.50); complete vision recovery (70% vs. 43%, *p* = 0.002) with a higher mortality signal (RR 2.15, 1.32–3.50). Evidence assessment scores high for principle feasibility in RCTs (9/10) and target-specificity in PLATO (9/10), identifying a critical gap in dedicated PCA RCT evidence.

**Conclusion:**

Endovascular therapy for acute PCA occlusion is physiologically plausible, shows promising direct signals, but lacks randomized confirmation. This synthesis suggests that EVT may be considered in severe cases but underscores the urgent need for a dedicated PCA thrombectomy RCT.

## 1 Introduction

Acute ischemic stroke is a leading cause of disability and mortality worldwide, with large vessel occlusion (LVO) representing its most severe clinical subtype (1). Supported by evidence from multiple landmark randomized controlled trials, endovascular therapy (EVT) has become the standard treatment for anterior circulation LVO (2). This successful paradigm has recently extended to the posterior circulation: the ATTENTION and BAOCHE trials demonstrated the efficacy and safety of EVT for acute basilar artery occlusion within 0–12 h and 6–24 h, respectively, thereby establishing the general principle of thrombectomy for posterior circulation LVO (3, 4).

However, a critical and unresolved question arises within this established framework: is EVT equally applicable to occlusions of distal posterior circulation vessels, particularly the posterior cerebral artery (PCA)? Although relatively less common, PCA occlusion can lead to a distinct and disabling syndrome characterized by homonymous hemianopia, sensory deficits, and higher-order cognitive impairments, severely compromising patients’ quality of life (5, 6). Furthermore, existing evidence indicates that even with successful recanalization therapy, functional outcomes for patients with posterior circulation large vessel occlusion are often suboptimal, with a limited proportion achieving complete recovery (7). This prognostic challenge extends to, and may be particularly relevant for, occlusions of the PCA, where the clinical impact is predominantly defined by disabling deficits rather than survival. This distinction creates a critical evidence gap: robust data from dedicated randomized trials assessing this disability-prevention versus risk trade-off are currently absent.

Consequently, the evidence supporting EVT for PCA occlusion remains fragmented and insufficient to guide practice. This knowledge gap lies precisely between two established thrombectomy principles: one for anterior circulation LVO and the other, recently established, for posterior circulation basilar artery occlusion. Specifically, the existing evidence is compartmentalized: randomized controlled trials (ATTENTION/BAOCHE) provide high-level validation of the underlying principle for posterior circulation therapy, with their inclusion of basilar tip occlusion subgroups indirectly supporting the rationale for PCA treatment (3, 4). In contrast, the observational study (PLATO) offers direct target-specific data by comparing outcomes in isolated PCA occlusion (6). However, dedicated evidence—such as high-quality subgroup analyses or RCTs targeting PCA occlusion—that could definitively bridge these two layers remains scarce and is associated with a low certainty of evidence (8). This heterogeneity in evidence sources, types, and strength results in a lack of a unified and reliable basis for clinical decision-making.

Given this uniquely compartmentalized evidence landscape, a traditional systematic review or meta-analysis aimed at deriving a single pooled effect estimate is neither statistically appropriate nor conceptually informative. This is particularly relevant as the efficacy of thrombectomy for posterior circulation sites beyond the basilar artery remains uncertain (8), and recent meta-analyses focusing specifically on PCA occlusion have not demonstrated clear functional benefit for EVT over medical management, highlighting the persistent uncertainty in the evidence (9). The substantial heterogeneity in study designs (RCT vs. observational), populations (severe BAO vs. isolated PCA), and outcomes (mortality reduction vs. disability prevention) would render such an aggregate result meaningless, while obscuring the complementary roles of each evidence layer that our framework aims to elucidate.

Therefore, this study employed a pre-specified evidence synthesis to integrate data from the three pivotal studies—ATTENTION and BAOCHE (principle layer) and PLATO (target-specific layer). We aimed to test the hypothesis that the current evidence for EVT in PCA occlusion is structurally dissociated—where high-level principle validation coexists with, but is not integrated into, high-specificity target signals—thereby forming a “confirmatory gap” that necessitates a dedicated RCT. By mapping this evidence landscape and assessing its completeness across multiple dimensions, this synthesis seeks to provide the necessary framework to guide future research.

## 2 Methods

### 2.1 Study design

This study was a pre-specified secondary analysis of three pre-identified pivotal trials (ATTENTION, BAOCHE, and PLATO) selected *a priori* as representative of the main high-quality evidence currently available for endovascular therapy (EVT) in acute posterior cerebral artery (PCA) occlusion. The analytical framework was defined *a priori* to test the specific hypothesis that a critical evidence gap exists between established thrombectomy principles (from basilar artery occlusion trials) and target-specific confirmation for PCA occlusion. The framework structured the evidence into three complementary layers (principle-indirect, target-specific, and connecting subgroup data) and employed a six-dimensional tool to assess evidence completeness.

No systematic literature search was performed because these three trials were selected *a priori* as the pivotal studies that collectively provide the main high-quality evidence currently available for this research question (two randomized controlled trials for basilar artery occlusion and one large observational study for isolated PCA occlusion). The study is therefore not a systematic review; rather, it is a hypothesis-driven secondary analysis of pre-selected trials. Accordingly, the reporting does not claim adherence to PRISMA 2020 nor PROSPERO registration. The analytical plan was pre-specified before data extraction. A dated protocol is available as Supplementary Protocol.

### 2.2 Data sources and included studies

The data for this synthesis were derived from three pre-selected core studies forming the main body of the current evidence system. The ATTENTION and BAOCHE trials are multicenter randomized controlled trials (RCTs) that demonstrated the efficacy and safety of EVT for acute basilar artery occlusion within 0–12 h and 6–24 h, respectively, providing the highest level of principle-based evidence for posterior circulation thrombectomy. The PLATO study is an international multicenter observational cohort that directly compared outcomes between EVT and medical therapy in patients with isolated PCA occlusion, offering the most direct target-specific data. These three studies collectively form an evidence chain from principle validation (RCTs) to direct evaluation (observational study).

### 2.3 Data extraction and quality assessment

Data extraction was performed independently by two investigators, and the extracted data were cross-verified. From the full-text publications and Supplementary material of each study, the following data were systematically extracted: study characteristics, sample size, patient baseline features (noting the proportion of PCA-supply-related subtypes like basilar tip occlusion), primary efficacy outcomes (expressed as adjusted Risk Ratio [aRR] or adjusted Odds Ratio [aOR] with 95% confidence intervals), and key safety outcomes. For the RCTs (ATTENTION, BAOCHE), methodological quality was assessed using the Cochrane Risk of Bias tool (RoB 2); for the observational study (PLATO), the Newcastle-Ottawa Scale was applied.

### 2.4 Data analysis and integration framework

Our analysis focused on qualitative synthesis and tabulated comparison. Given the substantial heterogeneity among the three studies in design, population, and outcome measures, a quantitative meta-analysis to pool effect estimates was not performed, as it would be statistically inappropriate and would obscure the complementary roles of each study—precisely the relationships our qualitative synthesis aims to elucidate.

First, a conceptual framework describing the logical progression of evidence was constructed. Second, the core efficacy and safety effect sizes from each study were tabulated (Table 1) to allow clear comparison while avoiding inappropriate direct pooling of different effect measures. Finally, a six-dimension evidence completeness assessment tool (Table 2) was developed and applied, constructed based on the pre-specified analytical framework. This tool, scoring from 0 to 10 across domains including Principle Feasibility, Target Specificity, and Direct Evidence Strength, was used to visualize the complementary roles and specific deficits of each evidence layer. The completeness of the current evidence and its major gaps were delineated through narrative synthesis.

**Table 1:** Effect estimates from ATTENTION, BAOCHE, and PLATO studies.

Study	Effect measure type	Primary efficacy estimate (95% CI)	sICH effect (95% CI)	90-day mortality effect (95% CI)
ATTENTION (RCT)	aRR (90-d mRS 0–3)	2.06 (1.46–2.91)	5.00 (CI not reported in original) [RR]	0.66 (0.53–0.82)
BAOCHE (RCT)	aRR (90-d mRS 0–3)	1.81 (1.26–2.60)	5.18 (0.64–42.18) [RR]	0.75 (0.54–1.04)
PLATO (observational)	aOR (90-d ordinal mRS shift)	1.13 (0.85–1.50)	3.77 (1.82–7.83) [aOR]	2.15 (1.32–3.50)

For sICH, the original publication of ATTENTION did not report a confidence interval; only the point estimate is shown. For BAOCHE and PLATO, the reported CIs were taken directly from the original publications. Different effect measures (aRR vs. aOR) and different primary outcome definitions preclude direct numerical comparison across studies. The table simply reports each study’s original estimates. For ATTENTION and BAOCHE, the sICH and mortality estimates are risk ratios (RR); for PLATO, they are odds ratios (OR) from univariable analysis [see Nguyen et al. (6), Table 4]. All confidence intervals for efficacy outcomes and for mortality (ATTENTION/BAOCHE) were taken directly from the original publications; no recalculation was performed.

**Table 2:** Evidence completeness assessment of the three core studies.

Assessment dimension	ATTENTION	BAOCHE	PLATO	Synthesis
Principle feasibility	9	9	5	RCTs provide a strong foundational principle.
Direct evidence strength	5	5	8	PLATO offers a direct efficacy signal.
Target specificity	4	4	9	PLATO has the highest target specificity.
Safety data	7	7	8	Safety profiles are relatively well-documented.
Evidence level	9	9	5	RCTs constitute the highest level of evidence.
Clinical guidance	6	5	7	All provide a certain degree of guidance.

Scores range from 0 to 10, with higher scores indicating greater evidence strength in that dimension. These scores are exploratory and subjective; they represent the authors’ qualitative judgment based on the predefined rubric and are not intended as precise quantitative measurements (see Supplementary Table S1 for the full scoring rubric). This tool has not been validated.

## 3 Results

### 3.1 Study characteristics and baseline data

The basic characteristics of the three pre-specified core studies included in this synthesis are presented in Table 3. Both the ATTENTION and BAOCHE trials were multicenter RCTs, enrolling 340 and 217 patients with acute basilar artery occlusion within time windows of 0–12 h and 6–24 h, respectively. The PLATO study was a multicenter observational cohort that enrolled 1,023 patients with isolated PCA occlusion within a 0–24 h window. The studies differed in population characteristics: ATTENTION and BAOCHE enrolled exclusively East Asian populations, whereas PLATO included multinational cohorts from Europe and North America. Baseline neurological deficit severity also varied substantially. Patients in ATTENTION and BAOCHE had more severe strokes (median NIHSS 24 and 20, respectively), while patients in PLATO presented with milder deficits (median NIHSS 8). Rates of intravenous thrombolysis were only reported in BAOCHE (14–21%) and PLATO (40–46%). This information was not explicitly provided in the ATTENTION publication.

**Table 3:** Integrated characteristics and outcomes of the three core studies.

Feature	ATTENTION trial	BAOCHE trial	PLATO study
Study design	Multicenter RCT	Multicenter RCT	Multicenter observational cohort
Target population	Acute basilar artery occlusion	Acute basilar artery occlusion (6–24 h)	Isolated PCA occlusion
Time window	0–12 h	6–24 h	0–24 h
Total sample size	340	217	1,023
Thrombectomy/control	226/114	110/107	378/645
primary outcome	90-day mRS 0–3	90-day mRS 0–3	90-day Ordinal mRS Shift
Primary effect measure	aRR 2.06 (1.46–2.91)	aRR 1.81 (1.26–2.60)	aOR 1.13 (0.85–1.50)
*P*-value	<0.001	<0.001	0.41
Functional independence (mRS 0–2)	33% vs. 11%	39% vs. 14%	51% vs. 53%
Excellent outcome (mRS 0–1)	Not reported	Not reported	32% vs. 27%
Early neurological improvement (NIHSS↓ ≥ 2)	Not reported	Not reported	66% vs. 44%
Complete vision recovery	Not applicable	Not applicable	70% vs. 43%
Symptomatic intracranial hemorrhage	5% vs. 0%	6% vs. 1%	6% vs. 2%
90-day all-cause mortality	37% vs. 55%	31% vs. 42%	10% vs. 5%
Procedure-related complications	Not reported	11%	Not reported
Any intracranial hemorrhage	13% vs. 2%	8% vs. 3%	Not reported
Baseline median NIHSS	24 (IQR 15–35)	20 (IQR 15–29)	8 (IQR 5–12)
Intravenous thrombolysis	Not explicitly reported	14% vs. 21%	40% vs. 46%
Population characteristics	100% East Asian	100% Chinese	European and North American multi-national

mRS, Modified Rankin Scale; NIHSS, National Institutes of Health Stroke Scale; IQR, interquartile range; EVT, endovascular therapy. Unless otherwise specified, values are presented as percentages or median (IQR).

### 3.2 Comparison of primary efficacy outcomes

For the two indirect supportive RCTs (ATTENTION and BAOCHE), both demonstrated significant and consistent benefit for the primary efficacy outcome (90-day mRS 0–3), with adjusted risk ratios of 2.06 (95% CI 1.46–2.91) and 1.81 (95% CI 1.26–2.60), respectively. For functional independence (mRS 0–2), both ATTENTION and BAOCHE showed clear superiority of the thrombectomy group over the control group (33% vs. 11 and 39% vs. 14%, respectively).

For the target-specific PLATO study, the primary efficacy outcome (ordinal analysis of 90-day mRS) was not statistically significant, with an adjusted odds ratio of 1.13 (95% CI 0.85–1.50, *p* = 0.41). The proportions of functional independence (mRS 0–2) were similar between the EVT and medical management groups (51% vs. 53%). Regarding the PCA-specific outcome of complete vision recovery, PLATO reported a higher rate in the EVT group (70% vs. 43%, *p* = 0.002). However, this finding should be interpreted with caution due to potential selection bias (vision outcome data may not have been systematically collected for all patients), lack of adjustment for multiplicity (multiple outcomes tested), and possible differences in outcome ascertainment between groups. The visual recovery signal is promising but requires confirmation in a randomized design.

The detailed effect estimates for primary efficacy and safety outcomes from the three studies are presented in Table 1. Readers are cautioned that different effect measures (aRR vs. aOR) and different primary outcome definitions (90-day mRS 0–3 vs. ordinal mRS shift) preclude direct numerical comparison across studies. The table simply reports each study’s original estimates as published.

### 3.3 Analysis of safety outcomes

Safety outcomes are also summarized in Table 1. The risk of symptomatic intracranial hemorrhage (sICH) was increased in the thrombectomy arm across all three studies, with point estimates of 5.00 (ATTENTION), 5.18 (BAOCHE), and 3.77 (PLATO, odds ratio from univariable analysis). The pattern for 90-day all-cause mortality differed. ATTENTION and BAOCHE showed a significant mortality reduction with thrombectomy (risk ratios 0.66 and 0.75, respectively), whereas PLATO indicated a higher mortality signal in the thrombectomy group (risk ratio 2.15, 95% CI 1.32–3.50). Procedure-related complications were reported in detail only in the BAOCHE trial, with an incidence of 11%.

### 3.4 PCA occlusion features and evidence sources

Table 4 systematically summarizes the anatomical features directly related to PCA occlusion and the type of evidence provided by each of the three studies. Among the principle-establishing RCTs, 33% of patients in ATTENTION and 12% in BAOCHE had basilar tip or distal basilar artery occlusions—subtypes that directly affect the origin of the PCA. While this provides an indirect anatomical and physiological rationale for therapy, neither trial reported independent efficacy data for these subgroups. In direct contrast, the PLATO study exclusively enrolled patients with isolated PCA occlusion (100%) and provided detailed segmental distribution of occlusions (P1: 53%, P2: 41%, P3: 3%), constituting the most direct source of target-specific evidence.

**Table 4:** PCA occlusion features and evidence hierarchy analysis.

Dimension	ATTENTION trial	BAOCHE trial	PLATO study	Synthesis
PCA-relevant occlusion type	Basilar tip occlusion	Distal basilar artery occlusion	Isolated PCA occlusion	Covers from PCA origin to distal segments
Proportion in study	33% (EVT), 35% (Control)	12% (EVT), 22% (Control)	100%	Increasing target specificity
Independent efficacy data	Not separately reported	Not separately reported (insufficient sample)	Reported (primary study population)	Direct comparative evidence only from the observational study
Occlusion segment breakdown	Not applicable	Not applicable	P1:53%, P2:41%, P3:3%	Mainly P1/P2 segment occlusions
Evidence type	Principle RCT (Posterior Circulation)	Principle RCT (Extended Time Window)	Targeted Observational Study	Complementary principle and target evidence
Principle feasibility score	9/10	9/10	5/10	ATTENTION/BAOCHE provide strong principle basis
Direct evidence score	6/10	6/10	8/10	PLATO provides direct signal but lower level
Target-specificity score	4/10	4/10	9/10	Current evidence shows a trade-off between specificity and level
Evidence gap	PCA subgroup data not separately reported	PCA subgroup sample insufficient for analysis	Non-randomized design, risk of bias	Underscores the need for a dedicated PCA RCT

“PCA-relevant occlusion” refers to occlusions affecting the PCA origin (basilar tip/distal basilar) in the RCTs, and isolated PCA occlusion in PLATO. “Independent Efficacy Data” refers to reported comparative outcomes (e.g., aRR/aOR) specifically for the PCA-relevant subgroup within a trial. Principle Feasibility, Direct Evidence Strength, and Target-specificity scores are derived from a pre-specified evidence completeness assessment tool (range 0–10, higher is better). They quantify the complementary roles of the studies. Evidence Gap summarizes the key limitation preventing definitive conclusions for PCA occlusion from each study source.

### 3.5 Evidence completeness assessment and synthesis landscape

The results of the evidence completeness assessment across six pre-defined dimensions are detailed in Table 2. The assessment quantifies the complementary nature and structural gaps within the current evidence. ATTENTION and BAOCHE scored highest in Principle Feasibility (9/10) and Evidence Level (9/10) but low in Target Specificity (4/10). Conversely, the PLATO study led in Target Specificity (9/10) and Direct Evidence Strength (8/10), though its Evidence Level score was limited by its observational design (5/10). Safety data were relatively complete across all studies (scores 7–8/10). In summary, the evidence completeness assessment reveals a starkly contrasting scoring profile between the two RCTs (ATTENTION/BAOCHE) and the observational study (PLATO) across key dimensions such as principle feasibility, target specificity, and evidence level (Table 2).

## 4 Discussion

### 4.1 Structural dissociation and therapeutic goal mismatch: mapping the PCA evidence gap

Our pre-specified synthesis of the ATTENTION, BAOCHE, and PLATO studies systematically maps a unique and critical gap in the evidence base for endovascular therapy (EVT) in acute PCA occlusion (3, 4, 6). The analysis reveals that the current evidence is structurally compartmentalized. High-level validation of the thrombectomy principle for posterior circulation large vessel occlusion, established by the ATTENTION and BAOCHE RCTs, remains disconnected from the direct, target-specific efficacy and safety signals for isolated PCA occlusion provided by the PLATO observational study (3, 4, 6). This compartmentalization reflects a deeper therapeutic goal mismatch. The success of EVT for basilar artery occlusion is measured against mortality reduction and prevention of severe disability—a framework validated for life-threatening proximal occlusions (3, 4). In stark contrast, PCA occlusion predominantly presents with disability-specific deficits, such as homonymous hemianopia, where the therapeutic imperative shifts from mortality reduction to the prevention of persistent, quality-of-life-limiting neurological impairment (5, 10). Consequently, clinical decision-making is forced into an ill-fitting extrapolation, attempting to apply a risk–benefit calculus derived from one disease severity extreme to another. The evidence completeness scores (Table 2) crystallize this divide: high Principle Feasibility is disconnected from high Target Specificity. Bridging this “confirmatory gap” therefore requires more than a new RCT; it necessitates a paradigm shift from a one-size-fits-all thrombectomy model toward a precision medicine approach tailored to the distinct pathophysiology and patient-centered priorities of distal territory strokes (11).

Furthermore, the distinct functional impact of PCA occlusion varies substantially by occlusion segment. As shown in Table 4, PLATO reported that P1 occlusions (53%) and P2 occlusions (41%) predominate. P1 segment occlusions involve the thalamus via the thalamogeniculate and posterior thalamic arteries, often leading to severe deficits including altered consciousness, cognitive impairment, or sensory syndromes (5). In contrast, P2/P3 occlusions mainly cause visual field cuts without thalamic involvement. This anatomical-functional distinction carries direct implications for EVT decision-making. In patients with thalamic involvement, the potential benefit of restoring thalamic perfusion may be greater given the more disabling symptoms; however, the procedural risk (e.g., sICH) might also be higher due to deeper, more fragile perforators. Therefore, future trials and clinical practice should stratify by occlusion segment, and the current indirect evidence from basilar artery trials (ATTENTION/BAOCHE) may be more applicable to P1 occlusions (as basilar tip occlusions affect the PCA origin) than to more distal occlusions. The importance of occlusion location in PCA stroke outcomes has been increasingly recognized (8, 10).

### 4.2 Deconstructing the contrasting mortality signal: implications for trial design and clinical thresholds

A pivotal finding of this integration is the contrasting mortality signal across studies: a clear reduction with EVT in severe basilar occlusion (ATTENTION/BAOCHE) versus a potential increase in isolated PCA occlusion (PLATO). This contrasting pattern is primarily rooted in the vast difference in baseline risk between the two populations. For high-mortality basilar occlusion, the substantial absolute survival benefit dominates the risk equation. In lower-severity PCA stroke, however, the absolute risk of procedural complications (e.g., symptomatic intracranial hemorrhage, sICH) becomes disproportionately significant when weighed against the primary goal of preventing specific disability (12).

This contrasting mortality signal has profound implications for the design of a future definitive “PCA-ATTENTION” trial, demanding a fundamental reconceptualization. Importantly, the higher mortality point estimate in the PLATO EVT arm (RR 2.15, 95% CI 1.32–3.50) should be interpreted with caution given the observational design and potential confounding. Nevertheless, this signal cannot be ignored and requires explicit evaluation in a dedicated randomized controlled trial.

First, the primary endpoint must be redefined to capture domain-specific disability prevention relevant to PCA syndromes. The conventional mRS 0–2 is inadequately sensitive. The endpoint should shift toward an ordinal analysis of the full mRS (shift analysis), a hierarchical composite endpoint prioritizing vision recovery, or a validated vision-specific quality of life measure, aligning with the emphasis on patient-reported outcomes (13, 14). Second, sample size calculations must be powered for a clinically meaningful difference in this disability-focused outcome, not for mortality, while incorporating rigorous pre-specified safety monitoring to establish a new distal vessel occlusion safety benchmark (7). Finally, the consistent sICH signal across all studies underscores a class effect of procedural risk in the delicate posterior circulation (3, 4, 6). For the PCA, supplying vulnerable structures like the thalamus, the risk of reperfusion injury may be heightened (8). Therefore, the trial must standardize a gentle reperfusion strategy tailored to distal anatomy, which may include smaller-bore devices like those studied for posterior circulation DMVO (15). This shifts the core question from “whether to treat” to “how best to treat” in this specific vascular territory.

### 4.3 A framework for precision revascularization: the three pillars of future progress

To translate the current physiological promise into confirmed clinical efficacy, we propose that future research must be guided by a precision revascularization framework, built on three interdependent pillars. Pillar I is advanced biomarker-driven patient selection. Future trials must prospectively validate PCA-specific imaging criteria, defining the optimal “PCA mismatch” using perfusion imaging or collateral scoring to identify patients with significant salvageable tissue most likely to benefit, while avoiding futile intervention (16, 17). Pillar II is anatomy-tailored technical protocolization. A standardized, gentle reperfusion toolkit should be mandated, with techniques optimized for the P1, P2, and P3 segments to minimize vessel injury and distal embolization (15). Pillar III is the implementation of a patient-centric core outcome set. This must incorporate domain-specific functional recovery and quality-of-life measures, ensuring that the trial’s success is judged by metrics that truly matter to patients (14).

A future RCT designed around this integrated framework would not only answer the specific question of PCA occlusion but also serve as a methodological blueprint for evaluating EVT in other distal vascular territories (14). The strengths of this synthesis lie in its pre-specified, hypothesis-driven methodology which avoids the pitfalls of an inappropriate quantitative meta-analysis. A key limitation is the marked heterogeneity among source studies, which precluded pooled analysis but was intrinsic to our research question. This underscores the critical need for a collaborative, patient-level data meta-analysis of the ATTENTION, BAOCHE, and PLATO cohorts to explore effect modifiers like occlusion segment and collateral status (18). Furthermore, the generalizability of findings is bounded by the enrolled populations, highlighting the need for inclusive future trials.

## 5 Conclusion

This prespecified evidence synthesis integrates data from the ATTENTION, BAOCHE, and PLATO studies to evaluate endovascular therapy for acute posterior cerebral artery occlusion. The current evidence is characterized as “established feasibility yet unconfirmed efficacy.”

The physiological rationale for EVT in PCA occlusion receives indirect support from high-level RCTs on basilar artery occlusion, and direct observational signals from PLATO—particularly for visual recovery—are encouraging but not definitive. However, these evidence components remain structurally disconnected, and no direct randomized confirmation currently exists.

Given the absence of definitive evidence, EVT for acute PCA occlusion should not be considered a standard treatment. For highly selected patients, it may be considered as an individualized treatment option after careful discussion of the potential benefits (e.g., visual recovery) and risks (e.g., procedural complications, a possible mortality signal as suggested by PLATO). Any such decision requires a thorough weighing of the available indirect and observational data.

A dedicated, adequately powered randomized controlled trial focusing exclusively on PCA occlusion is urgently needed to determine whether EVT provides a net benefit. Until such evidence is available, clinical practice should remain cautious and guided by individual patient circumstances.

## Data Availability

The original contributions presented in the study are included in the article/Supplementary material, further inquiries can be directed to the corresponding author.
