# Supplementary material for: Endovascular recanalization for acute posterior cerebral artery occlusion: a pre-specified secondary analysis of the ATTENTION, BAOCHE, and PLATO studies
Source: Front Neurol. 2026 Jul 3;17:1820036. doi: 10.3389/fneur.2026.1820036 (PMC13376863; doi:10.3389/fneur.2026.1820036)
Supplement: Supplementary file 1 [file Table_1.DOCX]

## Supplementary Table S1a. Scoring Rubric for Principle Feasibility, Direct Evidence Strength, and Target Specificity

| Dimension | Score Range | Criteria and Examples |
| --- | --- | --- |
| **Principle Feasibility** | 0‑3 | Very weak or no theoretical/physiological principle supporting EVT in posterior circulation. Example: Only preclinical or mechanistic studies available. |
|  | 4‑6 | Indirect evidence available (e.g., from basilar artery trials) but not directly applicable to PCA occlusion. Example: High‑quality RCTs on basilar artery occlusion with no PCA subgroup data. |
|  | 7‑10 | Strong principle established by RCTs that include PCA‑relevant subgroups (e.g., basilar tip occlusion). *Example: RCTs with reported PCA‑relevant subgroup but not as primary endpoint; 10 = dedicated PCA RCT with definitive results.* |
| **Direct Evidence Strength** | 0‑3 | No direct comparative data for isolated PCA occlusion. Example: Only case reports or no controlled comparison. |
|  | 4‑6 | Direct data available but from observational or non‑randomized studies. Example: Large prospective cohort with adjustment but not randomized (e.g., PLATO). |
|  | 7‑10 | Direct evidence from randomized controlled trials. *Example: RCT with PCA occlusion as a subgroup; 10 = well‑powered RCT designed specifically for PCA occlusion.* |
| **Target Specificity** | 0‑3 | No data specific to isolated PCA occlusion. Example: Mixes various distal posterior circulation occlusions. |
|  | 4‑6 | Partially specific data (e.g., includes basilar tip occlusions that affect PCA origin). Example: RCTs with a proportion of patients having PCA‑relevant occlusions but not isolated. |
|  | 7‑10 | Data entirely specific to isolated PCA occlusion. Example: Study exclusively enrolled isolated PCA occlusion patients (e.g., PLATO). |

**Scoring process:** Two authors independently assigned scores for each dimension based on the criteria above. Disagreements were resolved through discussion or by consulting a third author.

**Note:** This tool is exploratory and not validated; scores should be interpreted as qualitative guidance, not quantitative measurements. For scores falling within the same range (e.g., 4‑6), the exact integer was determined by the authors’ qualitative synthesis after discussion, considering ancillary factors such as sample size, confounder adjustment, and result consistency. This tool is not meant for precise quantitative measurement.

## Supplementary Table S1b. Scoring Rubric for Safety Data, Evidence Level, and Clinical Guidance

| Dimension | Score Range | Criteria and Examples |
| --- | --- | --- |
| **Safety Data** | 0‑3 | No safety outcomes reported. Example: No mention of adverse events. |
|  | 4‑6 | Safety outcomes reported but incomplete (e.g., only symptomatic intracranial hemorrhage, no procedure‑related complications). Example: Standard safety metrics reported but without subgroup details. |
|  | 7‑10 | Comprehensive safety data from well‑designed studies, including subgroup analyses. Example: Detailed safety outcomes by treatment arm, with confidence intervals. |
| **Evidence Level** | 0‑3 | Case series, expert opinion, or low‑quality observational studies. Example: Single‑arm retrospective registry. |
|  | 4‑6 | Observational cohort studies with adjustment for confounders. Example: Large multinational prospective cohort (e.g., PLATO). |
|  | 7‑10 | Randomized controlled trials (RCTs). *Example: Well‑designed RCT with low risk of bias; 10 = multiple high‑quality RCTs.* |
| **Clinical Guidance** | 0‑3 | No clear clinical implication. Example: Purely descriptive, no actionable recommendation. |
|  | 4‑6 | Provides general guidance but with major limitations (e.g., indirect evidence, small samples). Example: Suggests EVT may be considered in highly selected patients, but acknowledges uncertainty. |
|  | 7‑10 | Provides clear, actionable guidance supported by direct evidence. Example: Defines specific patient selection criteria and treatment algorithm. |

**Scoring process:** Two authors independently assigned scores for each dimension based on the criteria above. Disagreements were resolved through discussion or by consulting a third author.

**Note:** This tool is exploratory and not validated; scores should be interpreted as qualitative guidance, not quantitative measurements. For scores falling within the same range (e.g., 4‑6), the exact integer was determined by the authors’ qualitative synthesis after discussion, considering ancillary factors such as sample size, confounder adjustment, and result consistency. This tool is not meant for precise quantitative measurement.
