# Supplementary material for: Endovascular recanalization for acute posterior cerebral artery occlusion: a pre-specified secondary analysis of the ATTENTION, BAOCHE, and PLATO studies
Source: Front Neurol. 2026 Jul 3;17:1820036. doi: 10.3389/fneur.2026.1820036 (PMC13376863; doi:10.3389/fneur.2026.1820036)
Supplement: Supplementary file 3 [file Table_3.docx]

**Supplementary Protocol for Secondary Analysis of ATTENTION, BAOCHE, and PLATO Studies**

**Date:** 15 February 2026 (pre‑specified before data extraction)
**Study title:** Endovascular Recanalization for Acute Posterior Cerebral Artery Occlusion: A Pre‑specified Secondary Analysis of the ATTENTION, BAOCHE, and PLATO Studies
**Authors:** Xiaomin Xue, Ziqiang Yu, Baichen Wang

### **1. Objective**

To evaluate the evidence for endovascular therapy (EVT) in acute posterior cerebral artery (PCA) occlusion by integrating data from three pivotal studies:

- ATTENTION (basilar artery occlusion, 0‑12 hours)
- BAOCHE (basilar artery occlusion, 6‑24 hours)
- PLATO (isolated PCA occlusion, 0‑24 hours)

The analysis aims to characterize the structural dissociation between principle‑level and target‑specific evidence.

### **2. Pre‑specified analytical framework**

**Evidence layers:**

- **Indirect principle support:** ATTENTION and BAOCHE (RCTs for basilar artery occlusion).
- **Direct target‑specific data:** PLATO (observational cohort for isolated PCA occlusion).
- **Connecting subgroup data:** proportion of basilar tip/distal basilar occlusions in the RCTs.

**Primary outcomes:**

- 90‑day mRS 0–3 (ATTENTION/BAOCHE)
- Ordinal mRS shift (PLATO)
- Complete vision recovery (PLATO) as a PCA‑specific outcome

**Safety outcomes:**

- Symptomatic intracranial hemorrhage (sICH)
- 90‑day all‑cause mortality
- Procedure‑related complications

**Effect measures:**

- aRR for RCTs (ATTENTION, BAOCHE)
- aOR for PLATO

**No meta‑analytic pooling** will be performed due to cross‑trial heterogeneity in design, population, and outcome definitions.

**Evidence completeness assessment:**
Six‑dimensional tool (Principle Feasibility, Direct Evidence Strength, Target Specificity, Safety Data, Evidence Level, Clinical Guidance). Each dimension scored 0‑10 based on pre‑defined criteria (see Supplementary Tables S1a and S1b). The tool is exploratory and not validated.

### **3. Pre‑specified statistical approach**

1. No quantitative meta‑analysis will be conducted.
2. Results will be presented as individual study estimates with 95% confidence intervals.
3. All effect estimates and CIs will be extracted directly from the primary publications; no recalculation of CIs will be performed except when confidence intervals are not reported (e.g., sICH for ATTENTION/BAOCHE), in which case approximate intervals may be calculated using a continuity correction (these will be clearly identified).

### **4. Data extraction**

Two investigators will independently extract all data from the full‑text articles and online supplements. Disagreements will be resolved by discussion. Extracted data will be cross‑verified.

### **5. Protocol registration**

This analysis is not a systematic review and therefore is not eligible for PROSPERO registration. The protocol was documented in writing on the date above and is provided as supplementary material.

### **6. Deviations from protocol**

None.
